# Case report: Acute severe hyponatremia-induced seizures in a newborn: a community-acquired case and literature review

**DOI:** 10.3389/fphar.2024.1391024

**Published:** 2024-06-17

**Authors:** Haiting Li, Xiyang Chen, Linlin Chen, Jie Li, Xixi Liu, Caie Chen, Dengpan Xie, Junhui Yuan, Enfu Tao

**Affiliations:** ^1^ Department of Neonatology and NICU, Wenling Maternal and Child Health Care Hospital, Wenling, Zhejiang Province, China; ^2^ Department of Science and Education, Wenling Maternal and Child Healthcare Hospital, Wenling, Zhejiang Province, China

**Keywords:** neonatal hyponatremia, seizures, newborn, amplitude-integrated electroencephalogram, case report

## Abstract

Severe neonatal hyponatremia represents a critical electrolyte imbalance with potentially severe neurological outcomes, a condition rarely documented in community-acquired, full-term newborns. This report underscores a unique case of a 23-day-old, previously healthy, full-term male neonate experiencing severe hyponatremia that precipitated seizures, underscoring the urgency of prompt recognition and intervention. The neonate presented with symptoms including vomiting, groaning, chills, fixed staring, and limb tremors. Critical findings upon admission encompassed hypothermia, hypotension, tachycardia, and tachypnea accompanied by significant weight loss. The clinical presentation was marked by dehydration, lethargy, weak crying, a fixed gaze, irregular breathing, and coarse lung sounds, yet a distended abdomen, hypertonic limb movements, and recurrent seizures were observed. Immediate interventions included establishing IV access, rewarming, mechanical ventilation, seizure management, volume expansion, dopamine for circulatory support, and initiation of empirical antibiotics. Diagnostic evaluations revealed a sodium ion concentration of 105.9 mmol/L, while amplitude-integrated electroencephalography (aEEG) detected pronounced seizure activity characterized by a lack of sleep-wake rhythmicity, noticeable elevation in both the lower and upper amplitude margins, and a sustained decrease in the lower margin voltage dropping below 5 μV, presenting as sharp or serrated waveforms. The management strategy entailed rapid electrolyte normalization using hypertonic saline and sodium bicarbonate, anticonvulsant therapy, and comprehensive supportive care, with continuous aEEG monitoring until the cessation of seizures. Remarkably, by the third day, the neonate’s condition had stabilized, allowing for discharge in good health 10 days post-admission. At a 16-month follow-up, the child exhibited no adverse neurological outcomes and demonstrated favorable growth and development. Our extensive review on the etiology, clinical manifestations, aEEG monitoring, characteristics of seizures induced by severe neonatal hyponatremia, treatment approaches, and the prognosis for seizures triggered by severe hyponatremia aims to deepen the understanding and enhance clinical management of this complex condition. It stresses the importance of early detection, accurate diagnosis, and customized treatment protocols to improve outcomes for affected neonates. Additionally, this review accentuates the indispensable role of aEEG monitoring in managing neonates at elevated risk for seizures. Yet, the safety and efficacy of swiftly administering hypertonic saline for correcting severe hyponatremia-induced seizures necessitate further investigation through medical research.

## Introduction

Neonatal hyponatremia, commonly encountered in hospitalized newborns, significantly impacts morbidity and mortality rates (Saba et al., 2024). Normal serum sodium levels in neonates span from 135 to 145 mmol/L, with values below 135 mmol/L defining hyponatremia (Greenberg et al., 2015), categorized as mild (130–134 mmol/L), moderate (125–129 mmol/L), or severe (<125 mmol/L) (Adrogué and Madias, 2023). A recent study reported a 4.3% prevalence of neonatal hyponatremia, predominantly in preterm neonates within hospital settings (Storey et al., 2019). However, in a study analyzing 5,550 pediatric admissions, the incidence of community-acquired hyponatremia was found to be more common (15.8%) than hospital-acquired (1.4%), with the majority of cases being mild to moderate in severity and severe instances being notably rare (Rius-Peris et al., 2022). The etiological spectrum of neonatal hyponatremia is diverse, including iatrogenic factors, abnormalities in the renal mineralocorticoid pathway, inappropriate antidiuretic hormone secretion (SIADH), acute renal failure, heart failure, and gastrointestinal conditions such as gastroenteritis (Mazzoni et al., 2019), congenital chloride diarrhea (Wedenoja et al., 2010; Cendal et al., 2021), and necrotizing enterocolitis (NEC) (Palleri et al., 2022). However, severe community-acquired hyponatremia in full-term neonates is notably rare (Abelian and Ghinescu, 2015; Bryłka et al., 2018).

Typically, neonatal hyponatremia manifests asymptomatically or with mild symptoms like nausea and vomiting due to the neonatal skull’s flexibility, which can accommodate cerebral edema more readily (Storey et al., 2019). Chronic hyponatremia with slightly or moderately low sodium levels often remains symptomless (Miller et al., 2023). Severe symptoms, including seizures and obtundation, occur with acute sodium level drops below 120 mmol/L, posing significant risks of brain herniation and death (Miller et al., 2023; Saba et al., 2024), due to rapid intracerebral water migration (Miller et al., 2023).

As a primary trigger for neonatal seizures, the severity and duration of hyponatremia are crucial prognostic factors (PaSi et al., 2019). Alarmingly, in infants under 6 months without other causative factors, hyponatremia accounts for 70% of seizures (Farrar et al., 1995). Therefore, it is crucial to identify and promptly treat severe neonatal hyponatremia. However, inappropriate management of severe neonatal hyponatremia can lead to more serious complications, such as osmotic demyelination syndrome (ODS) (Adrogué et al., 2022; Şorodoc et al., 2023), central pontine myelinolysis (Balaini and Mahesh, 2023), and rapid shifts in fluid balance, potentially causing cerebral edema or brain damage (Marcialis et al., 2011a), and even death (Adrogué et al., 2022; Şorodoc et al., 2023).

Herein, we detail a rare instance of severe community-acquired hyponatremia in a full-term neonate, which precipitated seizures but was effectively managed through immediate intervention. Additionally, we have conducted an exhaustive review of the literature on neonatal hyponatremia, examining its etiology, clinical manifestations, amplitude-integrated electroencephalography (aEEG) presentations, and therapeutic approaches. This extensive analysis provides critical insights for clinical practice, significantly advancing our comprehension and handling of this complex condition.

## Case description

A 23-day-old, previously healthy full-term male neonate was admitted to our pediatric clinic with a day-long history of unexplained vomiting, 3 hours of groaning and chills, and episodes of fixed staring and limb tremors, without diarrhea. The infant was exclusively breastfed, and the exact amount of breast milk consumed was unknown. The mother assumed that the infant’s lack of fussiness was due to being full from breastfeeding. Born at 40 1/7 weeks gestation via spontaneous vaginal delivery, he weighed 2875 g at birth with mildly contaminated amniotic fluid. There was no history of premature membrane rupture, placental anomalies, asphyxia, or maternal health issues.

**FIGURE 1:**
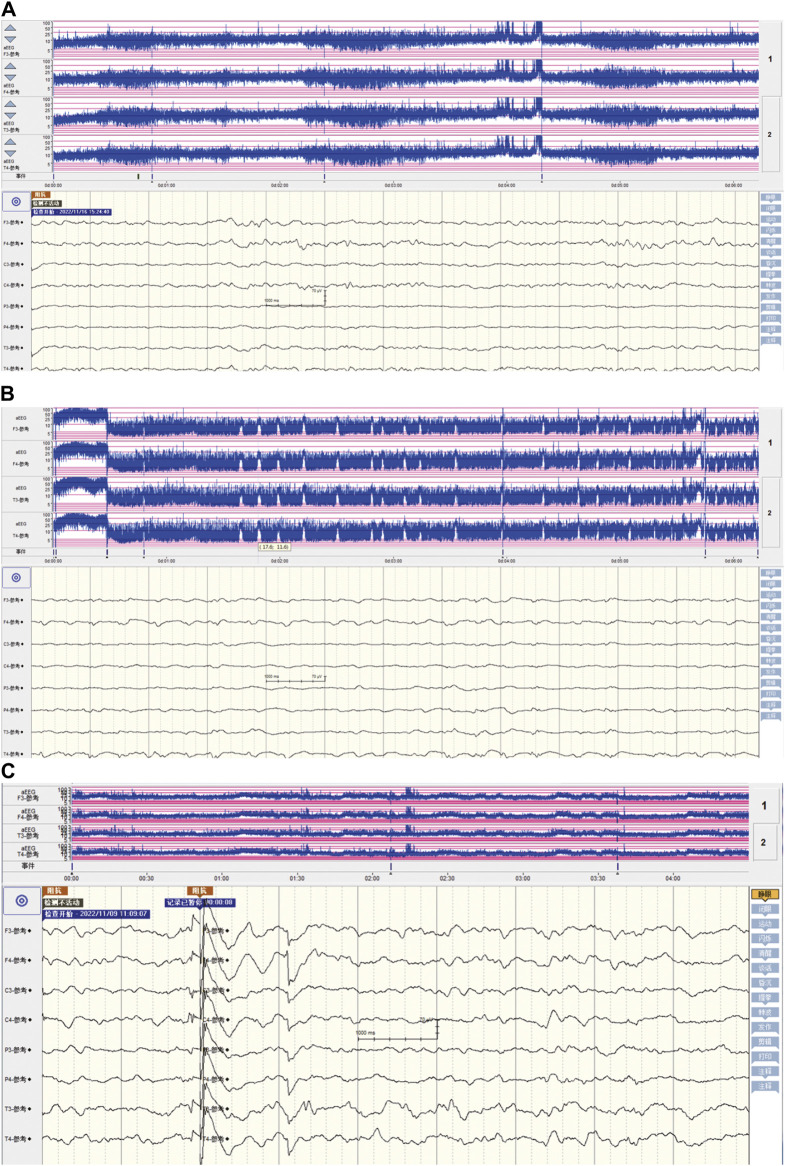
The characteristics of amplitude-integrated electroencephalography in neonatal seizures induced by community-acquired acute severe hyponatremia. **(A)** The aEEG presentation of a healthy full-term newborn. In a healthy full-term newborn, the aEEG trace typically fluctuates between wider and narrower patterns, reflecting periods of quiet sleep (QS) and periods of awake/active sleep (AS), respectively. During QS, the recording shows a more discontinuous pattern with the lower margin voltage (LMV) often dropping below 5 μV. Conversely, in AS and when the baby is awake, the LMV remains well above 5 μV. Regardless of the sleep state—AS or QS—the upper margin voltage (UMV) stays above 10 μV. This regular fluctuation is known as the “continuous normal voltage” (CNV) pattern and is generally straightforward for aEEG practitioners to recognize. **(B)** The aEEG patterns during severe hyponatremia-induced seizure episodes. The aEEG captured seizure activity characterized by the absence of sleep-wake rhythmicity, a pronounced elevation in both the lower and upper amplitude margins, with a sustained decrease in the lower margin voltage falling below 5 μV, manifesting as distinct sharp or serrated waveforms. **(C)** post-seizure control aEEG characteristics in community-acquired acute severe hyponatremia. After seizure management, the aEEG patterns have reverted to those observed in healthy full-term newborn.

**FIGURE 2:**
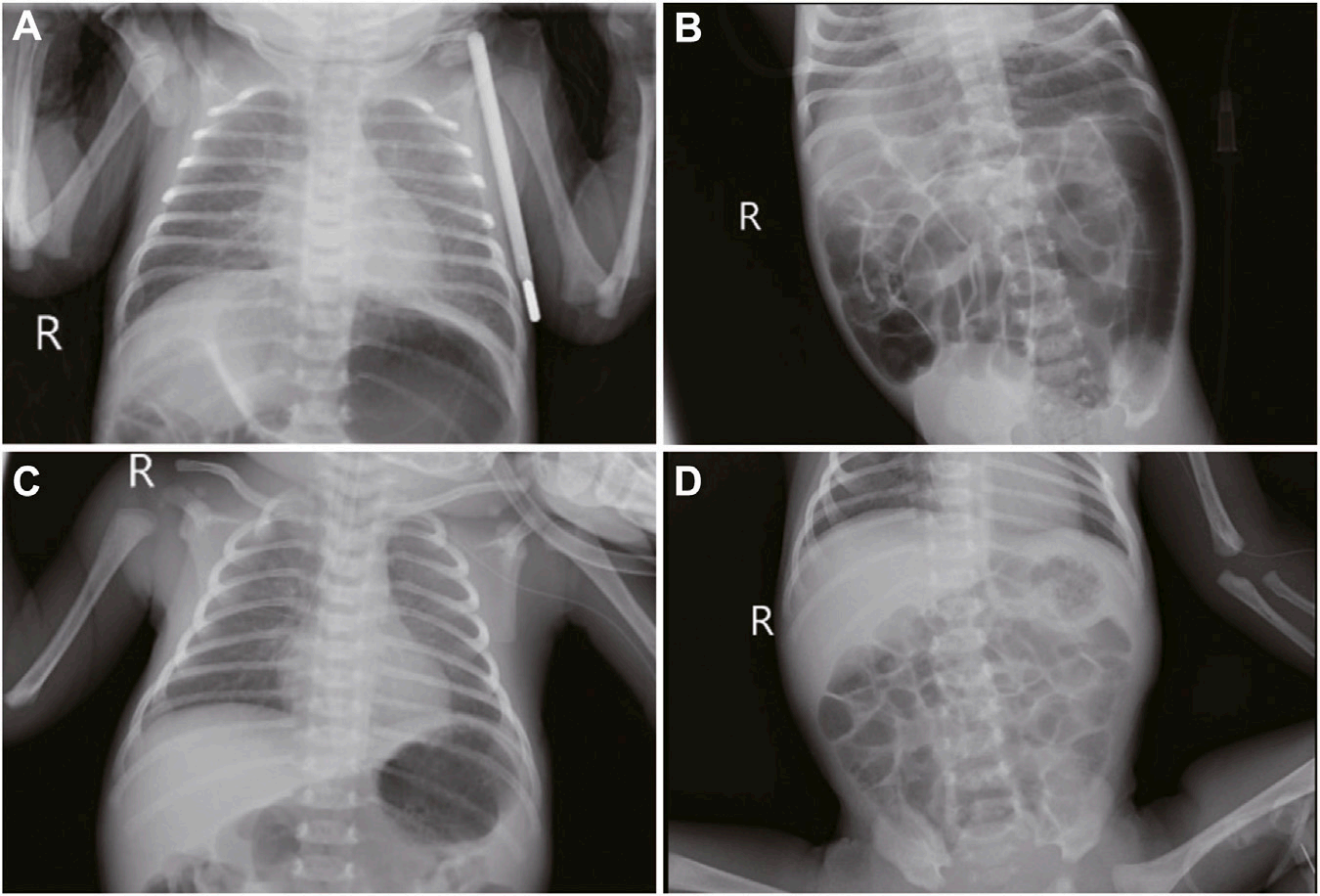
Imaging features in neonatal seizures induced by community-acquired acute severe hyponatremia. **(A, B)**: bedside chest and abdominal X-rays reveal scattered patchy and nodular densities along the pulmonary markings in the middle and lower lung fields bilaterally, presenting with blurred and poorly defined margins. There is a noticeable presence of gas within the colon. **(C)** chest and abdominal imaging under mechanical ventilation and peripheral central venous catheter. **(D)** abdominal imaging reexamination reveals a reduction in intestinal gas compared to previous assessment 2 days post-admission.

Upon admission, the neonate was in critical condition, presenting with hypothermia (below 35 °C), hypotension (42/26 mmHg), a pulse rate of 140 beats per minute, a respiratory rate of 52 breaths per minute, and a decreased weight of 2,610 g. Signs of dehydration, reduced responsiveness, weak crying, fixed gaze, and irregular breathing were noted. Cardiac and respiratory examinations were normal except for coarse lung sounds. The abdomen was distended without hepatosplenomegaly, and the limbs showed bicycling movements with increased tone. Frequent seizures characterized by breath-holding and limb twitching were observed, alongside reduced blood oxygen saturation. Emergency interventions included IV access, rewarming, mechanical ventilation, phenobarbital for seizures, saline fluid expansion, and dopamine for circulation support. Empirical antibiotic therapy (penicillin and cefotaxime) was initiated. Laboratory tests indicated a potential infection (white blood cell count of 14.2×10^9^/L, with 47.2% of neutrophils) and metabolic acidosis (pH of 7.13, base excess of −18.93 mmol/L, and actual bicarbonate of 8.5 mmol/L) with severe hyponatremia (sodium ion of 105.9 mmol/L), hypokalemia (potassium ion of 2.85 mmol/L), and hypochloremia (chloride ion of 84.7 mmol/L). The cerebrospinal fluid and cranial ultrasound showed no evidence of bacterial meningitis or intracranial hemorrhage, but aEEG confirmed seizure activity (Figure 1B; Supplementary Video S1). Bedside chest and abdominal X-rays reveal scattered patchy and nodular densities along the pulmonary markings in the middle and lower lung fields bilaterally, presenting with blurred and poorly defined margins, indicated neonatal pneumonia (Figure 2A). Additionally, there is a noticeable presence of gas within the colon (Figure 2B). The pyloric ultrasound revealed no notable abnormalities. Additionally, levels of both cortisol and 17-α-hydroxyprogesterone were within normal range.

**FIGURE 3:**
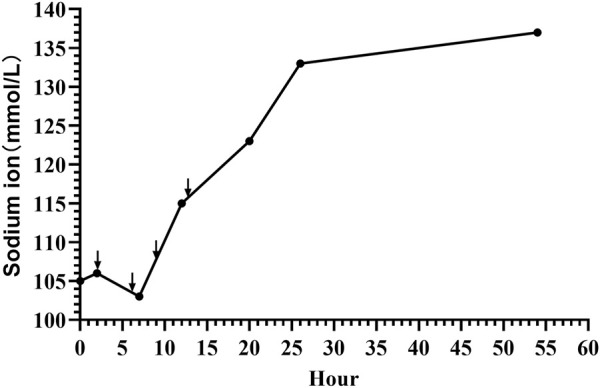
Sodium level dynamics in neonatal seizures induced by community-acquired acute severe hyponatremia. Black arrows mark the moments of 3% hypertonic saline supplementation, administered at 2, 6, 9, and 13 h post-admission.

The patient received 3% hypertonic saline and 1.4% sodium bicarbonate intravenously to correct hyponatremia and acidosis, respectively, with continuous electrolyte monitoring. To further alleviate the seizures, we administered a loading dose of phenobarbital (20 mg/kg) and subsequently midazolam at a rate of 1 μg/kg/min, employing both as anticonvulsants. Despite ongoing anticonvulsant treatment, the patient continued to experience intermittent seizures. We corrected the magnesium deficiency using magnesium sulfate and provided Vitamin B6 supplementation via a peripheral central venous catheter (PICC) (Figure 2C); however, the seizures persisted until the fourth administration of 3% hypertonic saline (Figure 3) successfully halted the convulsions (Figure 1C). In response to the infant’s marked weight loss and abdominal distension, parenteral nutrition was provided through PICC. By the second day after admission, notable improvements were observed in the patient’s condition; electrolyte levels had stabilized, seizures had ceased, allowing for the cessation of phenobarbital, and the necessity for ventilator support was eliminated. Blood and CSF cultures remained negative throughout, though C-reactive protein (CRP) levels peaked 5 days post-admission. The neonate continued to experience abdominal distension. Abdominal X-ray reexamination revealed a reduction in intestinal gas compared to previous assessment (Figure 2D). Antibiotic therapy was maintained until CRP levels returned to normal. The patient presented with a multifaceted clinical picture, diagnosed with conditions encompassing sepsis, septic shock, neonatal pneumonia, severe neonatal hyponatremia, hyponatremia-induced seizures, malnutrition, hypothermia, metabolic acidosis, hypochloremia, hypokalemia and hypomagnesemia. After 10 days of rigorous management, his weight increased to 2,730 g discharged in good health. One month after discharge, a cranial MRI was conducted in the outpatient setting, revealing no abnormalities. Upon follow-up at 16 months of age, the child was in good condition with no adverse neurological outcomes and demonstrated favorable growth and development. For a detailed overview of the key laboratory findings and special investigations undertaken during the diagnostic process were summarized in Table 1.

**TABLE 1:** Summary of laboratory investigations and special diagnostic procedures.

Investigation	Results	Reference range	Notes
Complete Blood Count white blood cell, ×10^9^/L	14.2	5–12	Highlights infection
neutrophil percentage, %	47.2	20–40	
lymphocytes percentage, %	45.4	50–75	
C-reactive protein, mg/L	<0.5, increased to a peak value of 30.8	0–5.0	Highlights infection
Blood gas			
pH	7.13	7.35–7.45	Suggests metabolic acidosis and tissue hypoxia
actual bicarbonate, mmol/L	8.5	18.0–26.0	
base excess, mmol/L	−18.93	−3.0∼+3.0	
lactic acid, mmol/L	6.7	0.5–2.2	
sodium ion, mmol/L	105.9	135–145	Indicates severe hyponatremia
potassium ion, mmol/L	2.85	3.5–5.5	Indicates hypokalemia
calcium ion, mmol/L	1.14	1.05–1.35	
chloride ion, mmol/L	101.9	98–113	Indicates hypochloridemia
blood glucose, mmol/L	2.9	3.9–6.1	
Blood biochemistry			
sodium ion, mmol/L	105	135–145	Indicates severe hyponatremia
potassium ion, mmol/L	4.39	3.5–5.5	
total calcium, mmol/L	2.65	2.25–2.75	
chloride ion, mmol/L	72	99–110	Indicates hypochloridemia
inorganic phosphate, mmol/L	1.92	1.45–2.1	
magnesium, mmol/L	0.68	0.75–1.02	Indicates hypomagnesemia
hepatorenal function	Normal	Normal	Suggests no hepatorenal involvement
Coagulation function	Normal	Normal	Excludes coagulopathy or disseminated intravascular coagulation
Urine routine test			
ketone bodies	1+	Negative	Indicates inadequate intake
urinary Crystal	Positive	Negative	Suggests dehydration
17α-hydroxyprogesterone, adrenocorticotropic hormone, and cortisol	Normal	Normal	Excludes primary adrenal insufficiency or congenital adrenal hyperplasia
Sputum and blood culture	Negative	Negative	Indicates no pathogen found
Thyroid function	Negative	Negative	Rules out thyroid-related causes
Cerebrospinal fluid examination	Negative	Negative	Suggests the absence of central nervous system infections
TORCH	Rubella virus, cytomegalovirus and herpes simplex virus type I IgG positive	Negative	Excludes TORCH infection, and IgG antibodies originates from the mother
Cranial ultrasound	Normal	Normal	Rules out intracranial hemorrhage
Gastric pyloric ultrasound	Normal	Normal	Excludes congenital hypertrophic pyloric stenosis
Abdominal ultrasound	low echogenicity within the gallbladder	Normal	Suggests bile stasis
Bedside chest and abdominal X-rays	scattered patchy and nodular densities along the pulmonary markings in the middle and lower lung fields bilaterally, presenting with blurred and poorly defined margins; noticeable presence of gas within the colon	Normal	Indicates neonatal pneumonia and rules out intestinal obstruction
Echocardiogram	open foramen ovale (φ 2.2 mm), patent ductus arteriosus (φ2mm), and mild tricuspid regurgitation	Normal	Exclues Cardiogenic shock
Amplitude integrated electroencephalogram	A sudden rise in both the lower and upper margins of the amplitude, followed by a brief amplitude depression, presenting as sharp or serrated patterns	Normal	Indicates neonatal seizures

TORCH: “T” stands for Toxoplasmosis, “O” for Other infections, “R” for Rubella, “C” for Cytomegalovirus (CMV), “H” for Herpes Simplex Virus.

## Discussion

We describe a unique case of seizures induced by severe hyponatremia, linked to malnutrition acquired in a community setting. This instance showcases the rare but critical phenomenon of convulsions resulting from significant hyponatremia. The patient’s favorable response to our timely and effective treatment led to a stable discharge. Nonetheless, this case highlights critical questions and considerations that merit deeper exploration and discussion.

Ensuring optimal short-term and long-term outcomes in neonates hinges on maintaining proper fluid and electrolyte homeostasis (Gordon et al., 2023; Segar and Jetton, 2023). Hyponatremia is notably common in neonates (Saba et al., 2024). The pathophysiological mechanisms of this condition are tied to the intake of water and sodium, as well as the regulation of these elements through renal filtration and urine production (Segar and Jetton, 2023). Vasopressin (VAP) serves as the primary regulatory hormone (Saba et al., 2024). Consequently, any disruption in the balance between water and sodium intake and excretion can lead to alterations in vasopressin secretion, potentially resulting in the development of hyponatremia. These factors include iatrogenic factors, fluid overload (Belik et al., 2001; Weaver et al., 2023), surgical stress response (Dogra et al., 2022), secondary pseudohypoaldosteronism (Kodo et al., 2021), diuretic use (Tan et al., 2020; Kalikkot Thekkeveedu et al., 2021), bacterial meningitis (Lin et al., 2016), community-acquired pneumonia (Guan et al., 2022), nutrition with fortified human milk alone (Gokçe and Oguz, 2020), feeding with breast milk (Kim et al., 2015) or donor human milk (Perrin et al., 2022), hypertrophic pyloric stenosis (Li et al., 2018), and congenital adrenal hyperplasia (Odenwald et al., 2016). Furthermore, malnutrition is also a significant cause of hyponatremia. Malnutrition can lead to hyponatremia by causing alterations in hydro-electrolytic balance, hormonal levels, inflammatory responses, or nutritional behavior changes (Baez et al., 2023). In this specific case, we posit that the primary cause of the severe hyponatremia was profound malnutrition, a consequence of inadequate nutritional intake. This is evidenced by the infant’s weight decline from a birth weight of 2,875 g–2,610 g at the onset of illness on the 23rd day of life, a clear indicator of significant malnutrition due to insufficient intake. Additionally, hyponatremia might result from excessive vomiting due to the loss of both fluids and electrolytes (Goren et al., 2016), and this infant presented to the clinic after experiencing vomiting for 1 day. Furthermore, inflammatory responses caused by infection could also be a significant factor leading to hyponatremia in this neonate. The most common cause of euvolemic hyponatremia is the syndrome of inappropriate antidiuretic hormone (SIADH) secretion, which can be significantly exacerbated by systemic inflammation. Additionally, CRP is associated with hyponatremia (Park and Shin, 2013; Baez et al., 2023). This infant exhibited elevated white blood cell count and CRP, indicating the presence of an infection-related inflammatory response. Despite this, both blood and cerebrospinal fluid (CSF) cultures showed no abnormalities. However, in cases of neonatal sepsis and even purulent meningitis, both blood and CSF cultures can yield negative results (Lin et al., 2016; Ge et al., 2021; Cantey and Prusakov, 2022). In summary, we believe that the severe hyponatremia observed in this case was due to insufficient intake, loss through vomiting, and infection.

Neonatal seizures are defined as seizures occurring within 4 weeks after birth in full-term infants or within 44 weeks of postmenstrual age in preterm infants (Yozawitz, 2023). The estimated incidence of these seizures is 2.29 cases per 1,000 live births, and higher rates were reported among preterm neonates than among full-term neonates (Yozawitz, 2023). Most seizures in neonates are acute provoked, as they occur as reactive events to acute insults, including hypoxic-ischemic encephalopathy (HIE), stroke, intracranial infection, genetic etiologies or transient metabolic disturbances, such as hypoglycemia or hyponatremia (Pressler et al., 2021; Carapancea and Cilio, 2023; Yozawitz, 2023). A prospective cross-sectional study conducted on 76 neonates admitted in tertiary level care NICU revealed that hyponatremia was manifested in 45 neonates (59.2%) with seizures, and out of those 45, 8 (17.7%) had severe hyponatremia, predominantly in 1–7 days (97.8%), only 2.2% in 8–28 days (PaSi et al., 2019). Notably, hyponatremia was present in 44 out of 62 neonates with HIE (PaSi et al., 2019). Hyponatremia is very common in moderate to severe HIE, especially in newborns who have undergone therapeutic hypothermia (Prempunpong et al., 2013). Numerous studies indicate that neonates with HIE exhibit the highest incidence of seizures (Weeke et al., 2015; Glass et al., 2016). It can be deduced that stemming from HIE plays a crucial role in the onset of neonatal seizures. However, it is imperative to note that substantiating this inference necessitates further empirical research. Given neonatal seizures caused by hyponatremia are more commonly observed during hospitalization (Modi and Edition, 1998), with community-acquired hyponatremia being quite rare, especially in severe cases. Our report expands the understanding of the etiology of seizures induced by neonatal hyponatremia, suggesting that more attention might be needed for electrolyte levels, particularly sodium levels, in newborns who are at home and vomiting after discharge.

The primary symptoms of hyponatremia are neurological, arising from reduced osmolality in the plasma, which initiates osmotic shifts of water into brain cells, subsequently resulting in cerebral edema (Moritz and Ayus, 2002). The clinical manifestations of hyponatremia primarily depend on the severity of the sodium deficiency and the rate at which hyponatremia develops (Marcialis et al., 2011b; Saba et al., 2024). Depending on the severity of hyponatremia, it can be classified as mild (130–134 mmol/L), moderate (125–129 mmol/L), or severe (<125 mmol/L) (Adrogué and Madias, 2023). Additionally, according to the speed of onset, it is classified into acute when occurring within 48 h and chronic occurring over 48 h or more or when the duration is unknown (Miller et al., 2023). In cases of chronic hyponatremia, mildly and moderately depressed sodium concentration may be asymptomatic (Miller et al., 2023). However, rapid changes in plasma sodium concentrations can cause severe, permanent, and sometimes lethal brain injury (Sterns, 2015). Brain cells possess the ability to self-regulate in order to maintain the homeostasis of brain cell volume (Gankam Kengne, 2023). This regulatory capacity of the brain primarily relies on the actions of water, electrolyte and organic osmolytes (Pasantes-Morales and Cruz-Rangel, 2010). Normally, extracellular osmolality equals intracellular osmolality (Gankam Kengne and Decaux, 2018). But with hyponatremia lowering extracellular osmotic pressure, water enters cells via aquaporins, causing brain cell swelling (Verbalis, 2010). In response to hyponatremia, brain cells activate regulatory volume decrease mechanisms to counteract cell swelling. Initially, cells release intracellular potassium and chloride, reducing ionic content to draw water out, minimizing volume increase. Subsequently, they extrude non-ionic osmolytes like betaine, taurine, and myo-inositol, a process possibly involving increased transporter activity (Gankam Kengne, 2023). However, these adaptive processes are time-sensitive and have limits. Thus, in cases of rapid or severe hyponatremia, these mechanisms may be insufficient, leading to cell swelling as water influx surpasses the rate of ion and osmolyte extrusion, potentially resulting in neurological symptoms (Gankam Kengne, 2023). Accordingly, brain swelling from an abrupt onset of hyponatremia results in increased intracranial pressure, impairing cerebral blood flow and sometimes causing herniation (Sterns, 2015). Moreover, in acute hyponatremia, adaptive release of glutamate, an excitatory neurotransmitter, may increase the susceptibility to seizures (Sterns, 2015). Symptoms of hyponatremia are more pronounced with acute hyponatremia where brain adaptation is incomplete while they are less prominent in chronic hyponatremia (Rondon-Berrios, 2023). Indeed, in our case, vomiting for 1 day led to acute hyponatremia, dropping blood sodium to 105.9 mmol/L and causing seizures. Contrarily, two other cases of chronic hyponatremia in premature infants, even with sodium as low as 95 mmol/L, did not exhibit seizures (Abelian and Ghinescu, 2015; Pérez-Pérez et al., 2023). This comparison suggests that in neonates, acute severe hyponatremia can manifest with seizures, while chronic severe hyponatremia may not show neurological symptoms despite sodium level is extremely low. It further indicates that the rate of hyponatremia onset might impact the nervous system more significantly than its severity.

Electroencephalography (EEG), and more specifically, video-EEG recording, is established as the gold standard in diagnosing neonatal seizures. (Pressler et al., 2021; Yozawitz, 2023). These seizures can manifest in two primary forms: electro-clinical seizures, where an electrographic seizure coincides with clinical signs, and electrographic-only seizures, which lack any apparent clinical symptoms (Pressler et al., 2021). The latter is notably prevalent in cases of HIE treated with therapeutic hypothermia (TH) (Nash et al., 2011). The significant occurrence of electrographic-only seizures in high-risk newborns, especially those with HIE undergoing TH, underscores the importance of continuous EEG monitoring. Timely detection and treatment of seizures are crucial as they may substantially improve outcomes (Kang and Kadam, 2015). In a study of 472 neonates, treatment with antiseizure medications within the first hour of seizure onset significantly reduced the overall seizure burden in the following 24 h compared to delayed treatment compared with those treated after 1 h of seizure onset (Pavel et al., 2022). Reversely, McBride et al. suggested that the extent of electrographic seizure activity in neonates, particularly those with perinatal asphyxia, is associated with increased risks of mortality and severe morbidity, indicating the potential benefit of more effective neonatal seizure management for improved neurodevelopmental outcomes (McBride et al., 2000). Additionally, continuous video-EEG monitoring is pivotal for diagnosing and managing neonatal seizures. A study showed that among 309 episodes suspected as seizures, only 20.4% were confirmed as clinical seizures via video-EEG. This led to alterations in antiepileptic drug regimens for 65.6% of the affected neonates, highlighting the critical role of accurate seizure diagnosis in guiding treatment decisions (Chan et al., 2022). Furthermore, in an instance of Kawasaki Disease complicated by Cerebral Salt-Wasting Syndrome, the patient did not experience seizures but displayed a decreased level of consciousness as the hyponatremia worsened. Nonetheless, electroencephalography indicated abnormal electrical activity consistent with acute encephalopathy. This underscores the importance of conducting EEG examinations in cases of hyponatremia (Oshima et al., 2020). We describe a case where severe hyponatremia resulted in convulsive seizures, with clinical signs of convulsions that were further substantiated by continuous video EEG monitoring. Notably, EEG details were absent in the documentation of two prior instances involving extremely severe hyponatremia in premature infants (Abelian and Ghinescu, 2015; Pérez-Pérez et al., 2023). It is advisable to implement EEG monitoring in such severe, non-convulsive cases to identify potential seizures early on. Initiating timely and appropriate treatment based on these observations may significantly contribute to enhancing patient prognosis (Abelian and Ghinescu, 2015; Pressler et al., 2023).

Clinically, seizures manifest in five primary forms: 1) motor, encompassing automatism, clonic, epileptic spasm, myoclonic, and tonic; 2) nonmotor, which includes autonomic and behavior arrest; 3) sequential; 4) electroencephalographic only; and 5) unclassified (Pressler et al., 2021; Spenard et al., 2024). A systematic review indicates a notable association between specific clinical seizure types and their underlying etiologies. For instance, autonomic seizures are predominantly linked to hemorrhage, while clonic seizures are significantly associated with central nervous system (CNS) infections and stroke. Moreover, metabolic or vitamin-related disorders, along with inborn errors of metabolism, are strongly correlated with the occurrence of myoclonic seizures (Nunes et al., 2019). Moreover, there were also specific EEG patterns seen with certain etiologies: vascular disorders and electrolyte imbalance with focal ictal discharges, vitamin-related disorders with multifocal, and all categories of genetic disorders with burst-suppression (Nunes et al., 2019). However, there are few reports on the seizure manifestations and electroencephalographic features induced by severe neonatal hyponatremia (Valerio et al., 2015). It had been reported that seizures in severe and rapidly evolving hyponatremia are usually generalized tonic-clonic (Nardone et al., 2016). Hyponatremia usually produces nonspecific EEG slowing. A very severe hyponatremia may initially cause posterior slowing followed by diffuse delta activity (Nardone et al., 2016). In fact, EEG demonstrates limited specificity in distinguishing between various electrolyte disturbances, such as hyponatremia, hypocalcemia, hypomagnesemia, and others (Nardone et al., 2016). However, it remains unclear whether there are specific EEG patterns associated with seizures or EEG abnormalities caused by acute, symptomatic severe neonatal hyponatremia, or in cases of chronic severe hyponatremia without clinical symptoms. This uncertainty persists due to the current lack of reports in this area. In our case study, the full-term infant exhibited mild myoclonic jerks of the limbs, with each episode lasting from approximately 10–30 s. Therefore, in our case of convulsions caused by acute severe hyponatremia, the clinical type of seizure is identified as myoclonic seizures (sudden, brief, irregular limb contractions) (Pressler et al., 2021; Yozawitz, 2023; Spenard et al., 2024). The aEEG with concurrent continuous video monitoring confirmed the presence of seizures (Figure 1B).

The aEEG of the infant revealed seizure characteristics marked by the cessation of sleep-wake rhythmicity, an abrupt increase in the amplitude’s lower and upper margins, followed by a sustained low margin voltage dipping below 5 μV. These episodes were characterized by distinct sharp or serrated waveforms. Our case clearly illustrates the seizure manifestations and aEEG patterns associated with acute severe hyponatremia in term infants, highlighting a notable instance of such characteristics in neonates with community-acquired acute severe hyponatremia. This contribution offers critical clinical insights for the management of similar cases, emphasizing its significance in the broader context of neonatal care.

The treatment of acute severe hyponatremia involves multiple aspects. The primary focus is on stabilizing cardiopulmonary function, promptly correcting hyponatremia, and administering anticonvulsant therapy, while actively searching for the underlying cause (Yozawitz, 2023). In the state of hyponatremic seizures, breathing may be easily suppressed, leading to a drop in blood oxygen saturation and potentially resulting in sudden death of the patient (Sainju et al., 2023). From a mechanistic standpoint, acute severe hyponatremia decreases cerebral blood flow and arterial oxygen content, which, when compounded by systemic hypoxemia, severely impairs the brain’s capacity to adapt to the low sodium levels. This impaired adaptation triggers a cascade of events leading to hyponatremic encephalopathy, which can manifest suddenly as respiratory failure due to mechanisms such as non-cardiogenic pulmonary edema or hypercapnic respiratory failure (Moritz and Ayus, 2003). Moreover, subsequent discussions will further highlight that the administration of anticonvulsant medications can also contribute to adverse effects like respiratory depression and hypotension (Qiao et al., 2021). It aptly underlines the critical role of cardiopulmonary management in addressing hyponatremic seizures. In our case, the neonate’s recurrent seizures caused apnea and reduced oxygen saturation, necessitating immediate intervention with invasive mechanical ventilation. The next focus of treatment is on swiftly correcting hyponatremia to halt seizures while avoiding serious adverse reactions from overly rapid sodium correction. The management of acute severe hyponatremia is currently treated with utmost prudence (Sterns et al., 2023). Three percent sodium chloride is commonly used to normalize or augment serum sodium level in terms of severe hyponatremia (Jannotta et al., 2021). Severely symptomatic hyponatremia is an urgent condition, and US and European guidelines recommend bolus hypertonic saline to cautiously elevate serum sodium by 4–6 mEq/L within 1–2 h, not exceeding 10 mEq/L in the first 24 h to mitigate hyponatremic encephalopathy (Adrogué et al., 2022). A recent review scrutinized the existing body of evidence and concluded that, despite some criticisms regarding the conservative nature of current hyponatremia correction guidelines, adherence to these protocols is advisable pending the emergence of further substantiated evidence (Sterns et al., 2023). However, a multicenter recent observational study indicated that limiting the sodium correction rate in patients with severe hyponatremia was associated with higher mortality and longer length of stay (Kinoshita et al., 2023). Michael L et al. recently suggested revising hyponatremia treatment guidelines to advocate a 15–20 mmol/L correction limit over 48 h, as rapid correction of severe hyponatremia may lead to improved outcomes (Moritz and Ayus, 2023). In line with this view, Sterns found that faster sodium replenishment in 10 patients with acute severe hyponatremia (sodium levels 101–106 mmol/L, replenishment rates 0.88–2.4 mmol/L/h) did not result in neurological sequelae. However, in a separate group of 54 patients with chronic severe hyponatremia (sodium levels 101–109 mmol/L, replenishment rates 0.41–0.66 mmol/L/h), slower sodium replacement led to neurological sequelae in seven patients, underscoring a significant difference in outcomes between acute and chronic conditions (Sterns, 1987). These findings emphasize the importance of distinguishing between acute and chronic severe hyponatremia, as the approach to sodium replenishment can significantly influence the outcome. For acute severe hyponatremia, rapid sodium replenishment can swiftly correct the low sodium levels, thereby preventing complications. However, for chronic severe hyponatremia, a more cautious approach to sodium replenishment is necessary, as rapid replenishment may lead to severe neurological sequelae. The difference in outcomes between the two conditions is related to the brain’s varying compensatory abilities in responding to acute and chronic severe hyponatremia, particularly the compensatory capacities of astrocytes. The foot processes of astrocytes, which encircle both brain capillaries and neurons, express aquaporins (such as aquaporin-4) that allow water to cross the blood–brain barrier (Day et al., 2014). Astrocytes protect neurons from osmotic stress; in response to hypotonicity, a cell-to-cell transfer of taurine to adjacent astrocytes allows neurons to maintain their volume while astrocytes swell (Verbalis, 2010). Within 24–48 h after this transfer, astrocytes restore their volume through loss of organic osmolytes, but this makes them vulnerable to injury from rapid normalization of the plasma sodium concentration. Because of the downregulation of transporters, recovery of lost brain osmolytes may take a week or longer (Pasantes-Morales and Cruz-Rangel, 2010; Verbalis, 2010). Therefore, rapid correction of hyponatremia is a hypertonic stress to astrocytes that are depleted of osmolytes, triggering apoptosis, disruption of the blood–brain barrier, followed by a loss of trophic communication between astrocytes and oligodendrocytes, secondary inflammation, microglial activation, and finally demyelination (Gankam Kengne et al., 2011). This represents a critical mechanism in chronic severe hyponatremia, where the excessively rapid correction of hyponatremia precipitates demyelinating processes. In cases of acute severe hyponatremia, the scenario differs. It is acknowledged that during such acute conditions, the brain, including astrocytes, lacks adequate time to fully adjust to the low sodium levels. Hence, rapid sodium correction is generally deemed safe and not commonly associated with negative impacts on astrocytes (Gankam Kengne, 2023; Rondon-Berrios, 2023). For acute severe hyponatremia signaling extensive cerebral edema, swift sodium correction is vital (Rondon-Berrios, 2023). American expert panel recommends increasing plasma sodium by 4–6 mmol/L in the initial hour (Verbalis et al., 2013), paralleling the European guideline’s 5 mmol/L rise (Spasovski et al., 2014). This 4–6 mmol/L elevation in plasma sodium in any 24 h period can critically reverse imminent brain herniation or cease seizures in such acute cases (Sterns et al., 2009; Sterns, 2015), and suggested therapeutic goals of 6–8 mmol/L in 24 h, 12–14 mmol/L in 48 h, and 14–16 mmol/L in 72 h (Sterns et al., 2009). The typical approach involves administering a hypertonic saline bolus, specifically 3–5 mL/kg of 3% NaCl, over a period of 10–15 min (Brown and Paloian, 2023). Serum sodium is estimated to increase by approximately 1 mmol/L with every 1 mL/kg of 3% NaCl (Moritz and Ayus, 2002). According to the latest guidelines from the International League Against Epilepsy (ILAE), phenobarbital is considered the first-line medication for controlling neonatal seizures, regardless of the cause. Additionally, for cases where phenobarbital does not provide a response, phenytoin, levetiracetam, midazolam, or lidocaine may be employed as second-line treatments (Pressler et al., 2023). In our case, the neonate’s blood sodium level was 105.9 mmol/L. We initiated treatment with 3 mL/kg of 3% NaCl to correct the hyponatremia, but the seizures did not cease and continued frequently. Therefore, we administered a loading dose of phenobarbital (20 mg/kg) to further control the seizures. Despite this, the child continued to experience seizures, prompting us to add midazolam. It is reported that seizures caused by hyponatremia in infants are often challenging to control (Sharf, 1993). Indeed, even after our interventions, the aEEG still indicated seizure activity. Observing that the blood sodium levels did not notably rise, we adhered to the 2013 American expert panel recommendations (Verbalis et al., 2013) and 2014 European clinical practice guidelines (Spasovski et al., 2014) by administering two additional doses of 3% NaCl. Considering that hypomagnesemia can also cause neonatal seizures, we corrected the low magnesium level (Cappellari et al., 2016). In rare cases, pyridoxine deficiency (one form of Vitamin B6.) may lead to neonatal seizures (Ipek et al., 2022), so we supplemented with vitamin B6. Besides this, we did not identify any other potential causes for the neonate’s seizures. After rechecking the electrolyte levels, the blood sodium had reached 115 mmol/L, but the neonate still experienced seizures. Consequently, we opted to administer a fourth dose of 3% NaCl, aligning with the notion that serum sodium should be adequately increased to effectively mitigate the symptoms (Gankam Kengne, 2023). Finally, the seizures ceased, and a subsequent electrolyte check showed that the blood sodium level had risen to 124 mmol/L. Therefore, it indicates that the neonatal seizures of our case were due to acute severe hyponatremia. Furthermore, we observed in our case that elevating the blood sodium level by 18.1 mmol/L within the first 24 h was essential to successfully arrest the seizures. This far exceeds the currently recommended rate of correction for hyponatremia (Sterns et al., 2009; Verbalis et al., 2013; Spasovski et al., 2014; Sterns, 2015). However, this is consistent with the perspective on rapid sodium correction by Michael et al. (Moritz and Ayus, 2023) and aligns with the outcomes of rapid correction for acute severe hyponatremia as reported by Sterns (Sterns, 1987). Moreover, our swift intervention to correct hyponatremia and cease seizures did not lead to any severe complications, such as demyelination, brain herniation, or mortality. Hence, it may be justifiable to employ rapid sodium correction strategies for acute severe hyponatremia in neonates, particularly when aimed at halting seizure manifestations. The scarcity of such cases underscores the need for extensive, multicenter, and global collaborative research.

The outcome of seizures in newborns is influenced by factors such as the underlying cause, the age at which seizures begin, the duration of the seizures, and the effectiveness of the medication. Prompt identification and management are deemed crucial in minimizing the detrimental impacts of the seizures and in fostering improved long-term prognosis (Wusthoff et al., 2021). Additionally, the nature of convulsions, whether unprovoked or provoked, plays a pivotal role in determining the prognosis. (Pisani et al., 2021). Unprovoked neonatal seizures were commonly considered as the clinical manifestation of early onset structural or genetic epilepsies that often have the characteristics of early onset epileptic encephalopathies (Pisani et al., 2021), and have a poor overall prognosis (Yozawitz, 2023). On the contrary, most seizures in neonates are acute provoked, as they occur as reactive events to acute insults, including HIE, stroke, intracranial infection, or transient metabolic disturbances (Yozawitz, 2023). Unlike acute symptomatic seizures, which are triggered by acute illnesses such as stroke, CNS infections, or traumatic brain injury, provoked seizures result from transient and reversible alterations without any structural changes. These alterations can be due to factors such as toxins, metabolic imbalances, or medications (Gunawardane and Fields, 2018). Therefore, seizures induced by acute severe neonatal hyponatremia can be reversible, and their prognosis is largely determined by the timely correction of hyponatremia and the control of seizure episodes (Spagnoli et al., 2018). Indeed, untreated or prolonged neonatal seizures can cause brain injury and worsen the clinical outcome (Miller et al., 2002; Yozawitz, 2023). A recent study has highlighted that the prognosis for epilepsies starting in the neonatal period is poorer compared to acute provoked neonatal seizures. Furthermore, it identified status epilepticus as the most significant predictive factor for negative outcomes (Lugli et al., 2024). This underscores the critical importance of promptly controlling seizures. Furthermore, severely abnormal background EEG activity have reported an predictors of abnormal outcome (Pisani et al., 2008). In our case, the favorable prognosis can be attributed to several key factors. Firstly, the seizure was provoked that was caused by hyponatremia, despite the severity. Secondly, the seizure was effectively managed within 24 h, following the fourth administration of 3% hypertonic saline. Thirdly, the normal background aEEG, serving as an encouraging sign for prognosis. Certainly, this is merely a case report, and further research is required for confirmation.

## Conclusion

We successfully treated a rare case of community-acquired acute severe neonatal hyponatremia with a favorable prognosis and no adverse neurological outcomes. Our comprehensive review deepens our understanding of the disease’s etiology, clinical presentations, monitoring, and treatment strategies for severe neonatal hyponatremia, significantly enriching the existing body of knowledge and shaping future clinical practices. Furthermore, it highlights the critical value of aEEG monitoring in managing neonates at high risk for seizures. Although rapid sodium replenishment proved successful in our case, the safety and efficacy of swiftly administering 3% hypertonic saline to correct seizures induced by severe hyponatremia necessitate further exploration through medical research.

## Data Availability

The original contributions presented in the study are included in the article/Supplementary material, further inquiries can be directed to the corresponding author.
